# The non-linear association between ascending aorta diameter and risk of 12-month mortality in Chinese patients with heart failure: A retrospective cohort study

**DOI:** 10.3389/fcvm.2022.917325

**Published:** 2022-08-30

**Authors:** Jin Chen, Yuan-Lei Huang, Hui Huang, Tao Zheng, Guang-Zhi Cong

**Affiliations:** ^1^Department of Cardiology, Affiliated Hospital of Guizhou Medical University, Guiyang, China; ^2^Department of Cardiology, The First Affiliated Hospital of Guizhou University of Traditional Chinese Medicine, Guiyang, China; ^3^Heart Center and Cardiovascular Institute, General Hospital of Ningxia Medical University, Yinchuan, China

**Keywords:** ascending aorta diameter, heart failure, 12-month mortality, non-linear, Chinese patients

## Abstract

**Background:**

There is no conclusive proven link between ascending aorta diameter (AoD) and the risk of death from heart failure (HF). As a result, a retrospective cohort analysis was carried out to determine whether AoD is associated with 12-month mortality in Chinese HF patients.

**Methods:**

From January 2017 to March 2020, we collected data on 575 Chinese patients with HF. The exposure and outcome variables were baseline AoD and 12-month risk of mortality (all-cause + cardiac origin), respectively. Data on demographics, drug usage, clinical characteristics, recognized indicators of HF, and comorbidities were included as covariates. To investigate the independent relationships of AoD with the risk of 12-month death, binary logistic regression and two-piecewise linear models were utilized.

**Results:**

Our findings imply that there was a non-linear relationship between AoD and the risk of 12-month mortality. For the AoD range of 23 to 37, there was no association with the risk of cardiac mortality [odds ratio (OR) 0.78, 95% confidence interval (CI), 0.62–1.04]. In the AoD range of 37–49, however, the risk of 12-month cardiac death increased by approximately 70% for every 1 mm increase in AoD (OR 1.70, 95% CI, 1.13–2.55). When all-cause death was chosen as the outcome, the same outcome was shown.

**Conclusion:**

An AoD larger than 37 mm is a hazardous threshold for Chinese HF patients. Beyond this limit increased the risk of cardiac death by 70% for every 1 mm increase in AoD.

## Introduction

Heart failure (HF) has long been a public health issue (1). It is the only cardiovascular disease whose incidence and prevalence have been increasing globally (2). According to Hao et al. (3) the total population of individuals with HF in China over the age of 35 is approximately 13.70 million (weighted prevalence of 1.3%). In China, the prevalence of HF has increased by 44% since 2000, when it was 0.9%. High disability, high readmission, and high death are all characteristics of HF (4). It not only increases the financial load, but it also drains medical resources. According to epidemiological data from other countries, the 5-year mortality rate of HF is significantly higher than that of several cancers (5). In China, HF mortality has nearly doubled in the last decade. The 12-month mortality rate for patients with severe HF is 40–50%, and 15–25% for patients with mild-to-moderate HF (6).

Echocardiography has traditionally been employed as the first-line imaging modality for HF (7). It can help with HF diagnosis, therapy effect monitoring, and grading severity (8). One of the important markers of echocardiography is the ascending aorta diameter (AoD). It is useful for diagnosing aortic disease and determining surgical indications (9). Furthermore, a greater pulmonary artery diameter (PAD) to AoD ratio (P/A) is frequently associated with a poor HF prognosis (10). However, the evidence for a long-term connection between AoD and HF mortality was extremely poor. So far, only a few studies have mentioned the link between AoD and HF prognosis (10–12). Furthermore, these studies do not completely account for confounders and produce contradictory outcomes. More importantly, this data did not come from Chinese patients. Given China’s large population and echocardiography as the first-line imaging modality, investigating the relationship between AoD and HF outcome is critical.

In light of this, the purpose of this study is to investigate the relationship between AoD and the risk of 12-month death (cardiac and all-cause) in Chinese HF patients. This study will give clinical data and fresh insights into the relationship between heart morphological features and outcomes.

## Participants and methods

### Study population

We conducted a retrospective cohort study between January 7, 2017 and March 9, 2020. The current study included 575 adult patients (18 years or older) with a primary hospitalization discharge diagnosis of HF. In the cardiology centers of two tertiary hospitals in Guizhou Province, we collected clinical information from the hospital’s electronic medical record system in a non-selective and sequential manner. The “Chinese Heart Failure Diagnosis and Treatment Guidelines 2018” are used to diagnose HF patients (6). The guidelines state unequivocally that history, physical examination, laboratory tests, cardiac imaging, and functionality testing are used to diagnose and assess HF. At the time of presentation, the physician first rules out HF based on the patient’s history, physical examination of the ECG, and chest radiograph, and then confirms the diagnosis with a natriuretic peptide test and echocardiogram. For the final data analysis, patients with the following conditions were included in this study: (1) met the guidelines for the diagnosis of HF; (2) were followed up on for at least 12 months; (3) patients had an echocardiogram and documented the value of AoD; This study was authorized by the ethical committees of the Affiliated Hospital of Guizhou Medical University and the First Affiliated Hospital of Guizhou University of Traditional Chinese Medicine (TCM). Because this was a retrospective cohort study and the patients’ personal information was anonymized, informed consent was waived.

### Variables

#### Outcome variable

We considered 12-month cardiac death as the primary outcome. We also included 12-month all-cause mortality as the secondary outcome. We characterized cardiac death as death due to myocardial infarction, arrhythmia, or HF (13). Patients who had less than a year of follow-up but survived were excluded as left-censored data. Patients who died during follow-up (within 12 months) were documented as dead (*Y* = 1), whereas those who remained alive after 12 months were recorded as alive (*Y* = 0).

#### Exposure variable and measurement

In this study, echocardiographic AoD values at admission were used as exposure variables and recorded as a continuous variable (mm). The two hospitals involved in this study are among the largest general hospitals in Guizhou Province. The echocardiographers were all skillful and experienced. Furthermore, the echocardiographers did not know the patient outcome, nor did they know that the data would be used for clinical research purposes in the future.

A two-dimensional imaging PLAX image is used for measurement. The patient is positioned in the left lateral position, with the probe in the third or fourth rib space on the left side of the sternum, near to the sternum, pointing toward the right shoulder, and holding exhalation to show the ascending aorta. At end-diastole, the maximum diameter is measured using the edge-to-edge technique in the long axis of the vertical ascending aortic vessel in the tubular part of the ascending aorta above the aortic sinus. The measurements were taken three times and then averaged.

#### Covariates

The selection of covariates in this study was mainly based on previous literature (14–18) that also investigated risk factors for HF prognosis. On this basis, the researchers also combined their own clinical experience and finally determined the following variables as covariates:

(1) Demographic-related indicators: age and sex (female/male);

(2) comorbidities: hypertension (yes/no), diabetes (yes/no), chronic obstructive disease (COPD) (yes/no) and ischemic etiologies (coronary heart disease or myocardial infarction or ischemic stroke and transient ischemic attack) (yes/no), Heart Valve Diseases (yes/no), age-adjusted charlson comorbidities index.

(3) Drug variables: diuretic use (yes)/no), digoxin use (yes/no), statin use (yes/no), Sacubitril valsartan use (yes/no), Ras-blocker use (yes/no);

(4) HF markers: N-terminal pro-B-type natriuretic peptide (NT-proBNP), high-sensitivity troponin (hs-CTnT);

(5) Other: body mass index (BMI), New York Heart Association classification (NYHA) (II + III/IV), HF duration (0–1 years/1–3 years/3–5 years/ > 5 years), and HF classification [HF with reduced ejection fraction (HFrEF), HF with mid-range ejection fraction (HFmrEF), HF with preserved ejection fraction (HFpEF)].

It should be noted that: (1) Because NT-proBNP testing was performed in two different hospitals, we first did a natural logarithmic transformation of NT-proBNP and then performed Z score processing on the transformed values. (2) Because of the small sample size, we adjusted for the number of factors. Combining myocardial infarction, coronary heart disease, and ischemic stroke as ischemic causes is primarily based on the common pathogenesis of ischemia, whereas combining angiotension converting enzyme inhibitors, angiotensin II receptor blockers, and spironolactone into Ras-blockers is primarily based on the fact that they have similar pharmacological properties.

## Missing data addressing

In this study, the ratio of missing data did not exceed 5%. Therefore, multiple imputation was not used (19).

### Statistical analysis

Continuous variables are denoted by their mean standard deviation, while categorical variables are denoted by their percent. Chi-square tests for categorical data and *t*-tests for continuous data were used to analyze differences between AoD tertiles. We used multivariate logistic regression to evaluate the relationship between AoD and the 12-month risk of mortality. Unadjusted models, minimally adjusted models, and fully adjusted models are all shown. Given that our sample size is small in comparison to the number of covariates that needed to be adjusted (18 covariates), we only adjusted for covariates that changed the matched odds ratio (OR) by at least 10% when added to the basic model (non-adjusted model) and full model (18 covariates were adjusted for) (20).

We also discovered a non-linear connection between AoD and the probability of 12-month mortality (all-cause and cardiac). The inflection point of the curve is calculated using smooth curve fitting and the recursion approach. A two-piecewise linear regression model is then used to calculate the OR and 95% confidence interval (CI) on both sides of the inflection point.

We performed the following sensitivity analysis to confirm the study’s robustness: (1) We converted the AoD from a continuous to a categorical variable (tertile) and calculated P for trend to see if the AoD OR values were robust. (2) Because of the gender disparities in AoD, we performed a stratified analysis using gender as a stratified variable to examine the relationship between AoD and 12-month mortality in males and females. (3) We also utilized LVEF as a stratified variable to see if the link of AoD with 12-month mortality was consistent among LVEF-based HF classifications. (4) To determine whether the exclusion of patients with missing AoD and patients with left-censored data will result in selection bias, we examined the distribution of each covariate between patients with AoD-missing and non-missing data, as well as patients with left-censored and non-left-censored data.

All the analyses were performed with the statistical software packages R (The R Foundation)^1^ and EmpowerStats (X&Y Solutions, Inc., Boston, MA).^2^ *P*-values less than 0.05 (two-sided) were considered statistically significant.

## Results

### The baseline characteristics of Chinese heart failure patients

**FIGURE 1:**
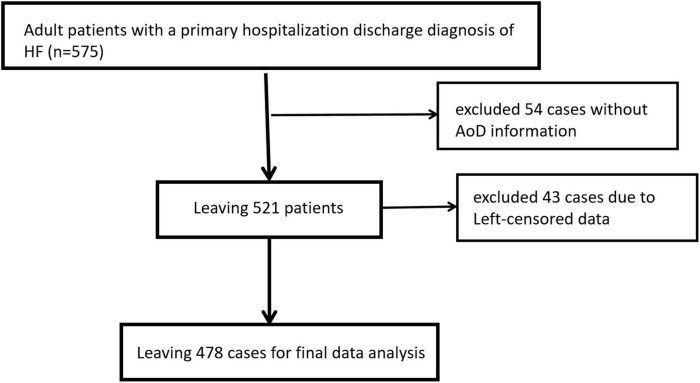
The flowchart of patients selection.

In the beginning, we collected data from 575 patients with a HF diagnosis from the hospital’s electronic medical record system. We then excluded 54 patients with missing AoD information and 43 patients with data that had been left censored. The remaining 478 patients were eventually used for final data analysis (Figure 1). With an average age of 72.06 ± 12.62 years, 230 (48.12%) of the 478 patients were male. Cardiac death and all-cause mortality at 12 months were 3.20% (15/469) and 5.02% (24/478), respectively. The distribution trends of patients’ baseline data among different AoD groups (According to tertile) are shown in Table 1. The low and middle AoD groups had a higher proportion of women, better cardiac function (fewer NYHA class IV patients), a higher proportion of patients with ischemic etiology, more digoxin use, a shorter HF burden, and more patients with HFpEF than the high AoD group.

**TABLE 1:** Baseline characteristics of patients with chronic heart failure.

Ascending aorta diameter (mm), tertile group	Low (23–26.00)	Middle (27.00–30.00)	High (31.00–49.00)	*P*-value
*N*	110	174	194	
Age, mean ± *SD*, year	72.49 ± 13.65	71.98 ± 13.19	71.89 ± 11.49	0.919
Ln Nt-Pro-BNP, mean ± *SD*, per 1 *SD* change	−0.14 ± 0.92	−0.03 ± 0.95	0.09 ± 1.05	0.142
Ln hs-Tnl, mean ± *SD*, per 1 *SD* change	−0.16 ± 0.97	0.06 ± 0.99	0.03 ± 1.03	0.172
BMI, mean ± *SD*, kg/m^2^	23.07 ± 3.62	23.19 ± 3.10	23.88 ± 3.94	0.083
aCCI, mean ± *SD*, score	1.90 ± 0.93	1.87 ± 0.91	1.81 ± 0.91	0.714
Sex, no (%)				<0.001
Female	80 (72.73%)	83 (47.70%)	85 (43.81%)	
Male	30 (27.27%)	91 (52.30%)	109 (56.19%)	
NYHA classification on admission, no (%)				0.008
II + III	102 (92.73%)	153 (87.93%)	156 (80.41%)	
IV	8 (7.27%)	21 (12.07%)	38 (19.59%)	
Comorbidity-hypertension, no (%)				0.19
No	28 (25.93%)	41 (23.98%)	61 (32.28%)	
Yes	80 (74.07%)	130 (76.02%)	128 (67.72%)	
Comorbidity-Diabetes, no (%)				0.608
No	82 (75.93%)	123 (71.93%)	144 (76.19%)	
Yes	26 (24.07%)	48 (28.07%)	45 (23.81%)	
Comorbidity-ischemic etiology, no (%)				0.002
No	35 (31.82%)	49 (28.16%)	87 (44.85%)	
Yes	75 (68.18%)	125 (71.84%)	107 (55.15%)	
Comorbidity-COPD, No (%)				0.056
No	100 (92.59%)	142 (83.04%)	167 (88.36%)	
Yes	8 (7.41%)	29 (16.96%)	22 (11.64%)	
Heart valve diseases, no (%)				0.025
No	90 (83.33%)	160 (93.57%)	169 (89.42%)	
Yes	18 (16.67%)	11 (6.43%)	20 (10.58%)	
Diuretics use, no (%)				0.018
No	35 (32.41%)	50 (29.24%)	36 (19.05%)	
Yes	73 (67.59%)	121 (70.76%)	153 (80.95%)	
Digoxin use, no (%)				0.118
No	90 (83.33%)	156 (91.23%)	169 (89.42%)	
Yes	18 (16.67%)	15 (8.77%)	20 (10.58%)	
Statins use, no (%)				0.133
No	31 (28.70%)	39 (22.81%)	61 (32.28%)	
Yes	77 (71.30%)	132 (77.19%)	128 (67.72%)	
Sacubitril valsartan use, no (%)				0.126
No	98 (89.09%)	148 (85.06%)	156 (80.41%)	
Yes	12 (10.91%)	26 (14.94%)	38 (19.59%)	
Ras blocker use, no (%)				0.275
No	29 (26.36%)	33 (18.97%)	38 (19.59%)	
Yes	81 (73.64%)	141 (81.03%)	156 (80.41%)	
Heart failure duration, No (%)				0.004
0–1 years	93 (86.11%)	139 (81.29%)	134 (70.90%)	
1–3 years	12 (11.11%)	15 (8.77%)	19 (10.05%)	
3–5 years	1 (0.93%)	10 (5.85%)	23 (12.17%)	
>5 years	2 (1.85%)	7 (4.09%)	13 (6.88%)	
Heart failure classification (by LVEF), no (%)				< 0.001
HFrEF (LVEF < 40%)	4 (3.64%)	26 (14.94%)	60 (30.93%)	
HFmrEF (LVEF 40–49%)	12 (10.91%)	15 (8.62%)	32 (16.49%)	
HFpEF (LVEF ≥ 50%)	94 (85.45%)	133 (76.44%)	102 (52.58%)	
12 month cardiac-cause mortality, no (%)				0.220
Survivor	102 (94.44%)	166 (98.22%)	184 (96.84%)	
Non-survivor	6 (5.56%)	3 (1.78%)	6 (3.16%)	
12 month all-cause mortality, no (%)				0.458
Survivor	102 (92.73%)	166 (95.40%)	186 (95.88%)	
Non-survivor	8 (7.27%)	8 (4.60%)	8 (4.12%)	

NT-proBNP, N-terminal pro B-type natriuretic peptide; Ln hs-Tnl, High-sensitivity troponin I; BMI, body mass index; ACCI, age-adjusted Charlson Comorbidity Index; NYHA, New York Heart Association; COPD, Chronic obstructive pulmonary disease; LVEF, left ventricular ejection fraction; HFrEF, heart failure with reduced ejection fraction; HFmrEF, heart failure with mid-range ejection fraction; HFpEF, heart failure with preserved ejection fraction.

### The association between aorta diameter and 12-month mortality obtained from univariate and multivariable logistic regression model

Table 2 displays the ORs and 95% CIs obtained from various models. The unadjusted model’s OR and corresponding 95% CI showed that there was no significant association between AoD and 12-month cardiac mortality (OR: 1.01, 95%: CI 0.91–1.13). Both the minimally adjusted (OR: 1.00, 95% CI: 0.90–1.12) and fully adjusted (OR: 0.98, 95% CI: 0.88–1.09) models yielded similar results. We also demonstrate the outcomes when AoD is used as a categorical variable (Tertiles). We discovered that the OR values in the middle and high AoD groups were not equidistant in all three models (Table 2). This finding strongly suggests that the relationship between AoD and cardiac mortality risk is likely non-linear.

**TABLE 2:** The results of univariate and multivariate analyses using binary logistic regression model.

Exposure	Non-adjusted OR, 95%CI	Minimally adjusted model OR, 95%CI	Fully adjusted model OR, 95%CI
AoD vs. 12-month cardiac mortality	1.01 (0.91, 1.13)	1.00 (0.90, 1.12)	0.98 (0.88, 1.09)
Low	Ref	Ref	Ref
Middle	0.31 (0.08, 1.26)	0.25 (0.06, 1.08)	0.53 (0.17, 1.63)
High	0.55 (0.17, 1.76)	0.45 (0.14, 1.51)	0.43 (0.09, 1.17)
*P* for trend	0.3779	0.2734	0.0867
AoD vs. 12-month All-cause mortality	0.99 (0.90, 1.08)	0.99 (0.90, 1.08)	0.97 (0.84, 1.11)
Low	Ref	Ref	Ref
Middle	0.88 (0.32, 2.39)	0.86 (0.31, 2.39)	0.20 (0.04, 0.96)
High	0.93 (0.34, 2.54)	0.90 (0.32, 2.53)	0.18 (0.03, 0.91)
*P* for trend	0.8856	0.8530	0.0423

Ref, reference.

95%CI: 95% confidence interval.

Non-adjusted model adjust for: None.

Minimally adjusted model adjust for: age, sex.

Fully adjusted model adjust for: Sex, NYHA classification on admission, ACCI, Heart Valve Diseases, diuretics use, digoxin use, statins use, sacubitril valsartan use, heart failure duration, Ln Nt-Pro-BNP (z-score), Ras blocker use, BMI, Heart failure classification.

Table 2 also shows the relationship between AoD and all-cause mortality in patients at month 12 of follow-up. Despite the change in definition, the results are nearly identical to those obtained when cardiac death was employed as an outcome. In the unadjusted (OR: 0.99, 95% CI: 0.90–1.08), minimally adjusted (OR: 0.99, 95% CI: 0.90–1.08), and fully adjusted models (OR: 0.97, 95% CI: 0.84–1.11), there was no significant link between AoD and all-cause death. Sensitivity analysis also suggested a non-linear relationship between AoD and all-cause mortality.

### The results of non-linearity of aorta diameter and 12-month mortality

**FIGURE 2:**
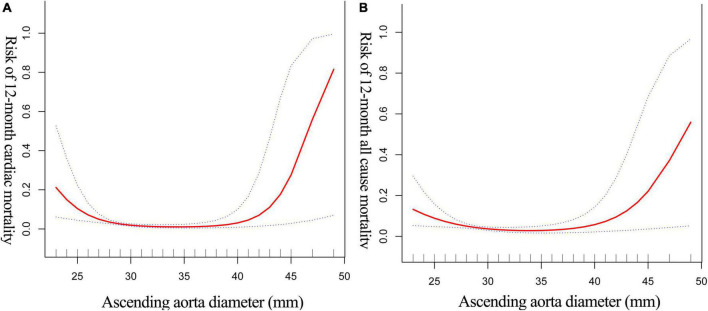
The non-linear relationship between AoD and 12-month cardiac-mortality **(A)** all-cause mortality **(B)** of HF. The abscissa represents the AoD. The ordinate represents the risk of 12-month mortality in patients with HF. The middle line represents the trend of 12-month mortality with AoD. The upper and lower lines represent the 95% confidence interval.

We investigated the non-linear relationship between AoD and the probability of death after 12 months in patients with HF. A smoothed curve fit (Figures 2A,B) revealed a threshold effect between AoD and both cardiac death and all-cause death. The recursive technique offers a 37-mm threshold. Table 3 shows the two-piecewise linear model findings. When cardiac death was used as the outcome variable, the results demonstrate that there was no connection between AoD and cardiac death to the left of the inflection point (AoD range: 23–37 mm) (OR: 0.78, 95% CI: 0.62–1.04). For individuals with HF, however, each 1 mm increase in AoD to the right of the inflection point (AoD range: 37–49 mm) was linked to a 70% higher risk of cardiac mortality (OR: 1.70, 95% CI: 1.13–2.55). When all-cause death was utilized as the outcome, the same results were obtained. Similarly, the inflection point was 37 mm, and there was no significant correlation between AoD and all-cause mortality to the left of the inflection point (OR: 0.90, 95% CI: 0.78–1.03). However, every 1 mm increase in AoD to the right of the inflection point increased the risk of all-cause death in HF patients by 31% (OR: 1.31, 95% CI: 1.01–1.76).

**TABLE 3:** Non-linearity addressing using two-piecewise linear model.

AoD vs. 12-month cardiac mortality	OR, 95%CI
Fitting using standard binary logistic regression model	0.98 (0.88, 1.09)
Fitting using two piecewise linear model	
Inflection point of ascending aorta diameter (mm)	37
23 to = 37	0.78 (0.62, 1.04)
>37 to 49	1.70 (1.13, 2.55)
*P* for log-likely hood ratio test	0.003
AoD vs. 12-month all-cause mortality	
Fitting using standard binary logistic regression model	0.97 (0.84, 1.11)
Fitting using two piecewise linear model	
Inflection point of ascending aorta diameter (mm)	37
23 to = 37	0.90 (0.78, 1.03)
>37 to 49	1.31 (1.01, 1.76)
*P* for log-likely hood ratio test	0.047

Covariates which were adjusted for: Sex, NYHA classification on admission, ACCI, Heart Valve Diseases, diuretics use, digoxin use, statins use, sacubitril valsartan use, heart failure duration, Ln Nt-Pro-BNP (z-score), Ras blocker use, BMI, Heart failure classification.

### The results of sensitivity analyses

We excluded 54 individuals during the enrollment process due to the lack of AoD. We did a sensitivity analysis to avoid any potential selection bias induced by these omitted patients. Our findings (Supplementary Table 1) revealed that there was no statistically significant variation in the distribution of baseline characteristics between patients with and without AoD (all *p*-values > 0.05).

We also excluded 43 patients with left-censored data. Our results (Supplementary Table 2) showed that these 43 patients were indistinguishable from the population with non-left-censored data in terms of baseline characteristics except for the HF duration.

### The results of stratified analyses

After discovering a non-linear relationship between AoD and the risk of mortality, we conducted a stratified analysis utilizing gender and type of HF as stratifying variables (see Supplementary Table 3 for details of the adjustment strategy). The goal of the stratified analyses was to determine whether the non-linear association between AoD and risk of mortality was consistent across gender and HF categories. Supplementary Figure 1 clearly demonstrates that the trends in the association between AoD and death (all-cause + cardiac) were extremely different between the sexes. The link between AoD and death in women is not statistically significant. In men, however, there is a strong trend toward a threshold effect (Supplementary Figures 1A,B). Through the use of a two-piecewise linear model, the results were validated. Due to the small sample size, the link between AoD and 12-month mortality was not found to be statistically significant (95% confidence interval across 1) (Supplementary Table 4), but the overall pattern was in line with what was seen in Supplementary Figures 1A,B.

Additionally, we used the type of HF as a stratification variable to divide the population into three groups: HFrEF, HFmrEF, and HFpEF. As seen in Supplementary Figures 1C,D, there was a stronger correlation between AoD and risk of mortality (all-cause + cardiac) in the HFrEF and HFmrEF populations. We combined patients with HFrEF and HFmrEF when verifying with a two-piecewise linear model since the trends in AoD were more similar in the HFrEF and HFmrEF populations. According to the findings (Supplementary Table 5), an increase in AoD above 37 mm was linked to a high risk of death in the HFrEF and HFmrEF populations.

In conclusion, while the final results are limited by sample size and do not allow for robust findings, the trend suggests that a non-linear relationship between AoD and death can be observed in men and patients with HFrEF and HFmrEF. Females and patients with HFpEF, on the other hand, had a flatter trend in AoD and death.

## Discussion

The current study investigated the relationship between AoD and 12-month mortality in Chinese HF patients. Our findings indicate that a positive relationship between AOD and 12-month mortality risk exists only when AOD exceeds a certain threshold (> 37 mm). According the results of the two-piecewise linear model, each 1 mm increase in AOD in the range 37–49 mm raises the risk of 12-month all-cause mortality by 31.0% and the risk of 12-month cardiac death by 70.0%.

Until this, a lot of work was invested in the “linear” correlation, but the results were inconsistent. Pellicori et al. (11) discovered no correlation between AoD and all-cause death in 384 HF patients (HR = 0.99, 95% CI 0.97–1.03). In 110 patients with severe stage HF, Chimura et al. (12) reported that the probability of adverse cardiovascular problems (heart-related death with implantation of a left ventricular assist device) increased by 20% for every 1 mm increase in AoD (HR = 1.20, 95% CI: 1.12–1.30). However, the two studies did not account for confounders, which significantly increase the risk of death. Furthermore, no studies have been published on the relationship between AoD and mortality in Chinese HF patients. In the current study, we found two distinct patterns of AoD with mortality (all-cause and cardiac) in different AoD ranges. These findings may be more representative of the actual clinical scenario than the linear relationship. Previous research has linked ascending aortic dilatation to increased aortic stiffness and left ventricular hypertrophy (21, 22). They are both independent risk factors for HF prognosis. As a result, higher mortality risk is only associated with ascending aortic dilatation (> 37 mm). This also explains previous studies’ inconsistency: Because the inclusion of patients in these studies was inconsistent and the proportion of patients with AoD was not accounted for, the association of AoD with HF events took on a completely different trend.

A series of stratified analyses were performed to investigate how these indicators might influence the differing impact of AoD on all-cause death risk. First, we stratified by gender (male vs. female) because AoD reference ranges differ by gender. We discovered that the threshold effect between AoD and 12-month death exists in males but not in females. Second, we classified patients based on their LVEF. The non-linear relationship between AoD and risk of all-cause death was found in patients with HF with midrange ejection fraction (LVEF 40–49%) and reduced ejection fraction (HFrEF; LVEF 40), but not in HF with preserved ejection fraction. As a result of stratified analyses, the non-linear relationship between AoD and 12-month mortality in this study appears to be detected in males and patients with HFrEF or HFmrEF.

In conclusion, our study has the following clinical significance: First, all of our patients were screened for AoD using ultrasound. Other, more accurate but also more expensive imaging devices (e.g., CT, etc.) were undeniably employed in other studies. However, we must consider China’s enormous number of HF patients. As a result, for the Chinese context and clinical scenario, an ultrasound-based evaluation of AoD and its connection with unfavorable cardiac outcomes would be more appropriate (cheaper and more generalizable). Second, while the current “normal” reference range for AoD in China is 23–35 mm, its applicability to HF patients remains debatable. We not only developed a risk “threshold” (37 cm), but we also established in our study the population to which this threshold applies (men with HFrEF and HFmrEF). Despite the fact that our stratified analysis was not robust in terms of sample size, this tendency will provide some clinical evidence for future studies.

Third, model-driven research is becoming increasingly popular in the cardiovascular sector. A rising variety of models and scores are emerging to improve cardiovascular treatment. Modeling, on the other hand, is based on determining the true relationship between each variable and the outcome. Forcing a linear fit for variables that are non-linear related to predicted outcomes might reduce model uncertainty significantly. Thus, our study provides at least some useful data material for subsequent model-driven class studies.

We list the advantages of our study: (1) in contrast to previous studies that were only univariate, we adjusted for variables associated with HF prognosis based on a literature search; and (2) the application of advanced algorithms (GAM model and two-piecewise linear model) allowed us to detect the true association of AoD with the risk of all-cause mortality in Chinese HF patients. (3) Stratified analysis found the association of AoD with all-cause mortality only in males and HF patients with LVEF greater than 40. This result helps us better understand the populations to which our findings may apply. There are also several limitations to this study. First, our study population is comprised of only Chinese HF patients. Therefore, extrapolation of our findings to other populations requires caution (e.g., non- HF patients and other races). Second, we can only adjust for measurable confounders, but not for unmeasured confounders. Third, because this study collected clinical information retrospectively, the measurer of echocardiography may be a confounding factor, as the measurer’s skill level may affect the accuracy of the measurement. However, the measurers couldn’t know that the study was going to be performed in the future at the time of measurement, which means that their measurements were independent of the patient’s prognosis. Although there may be measurement errors, such errors do not bias our findings. Fourth, we also found a threshold effect trend in the population with LVEF of less than 40%. However, the sample size of this subset of patients was too small to fail the model fit, making it impossible to determine whether our findings could be applied to HF patients with LVEF of less than 40%. Future prospective cohorts with larger samples are needed to validate the results. Fifth, in contrast to prospective cohort studies, retrospective cohorts collect data after the end of the follow-up period. They are so vulnerable to missing data and recall bias. However, (1) the baseline data for this study were obtained objectively in the electronic medical record system rather than through “recall”; (2) for the outcome variable (patient survival status), we combined community council, community hospital, and telephone follow-up to determine whether the patient had died; and (3) for the unavoidable missing data or lost follow-up that still exist, we assessed whether such missing data would cause bias (Supplementary Tables 1, 2). Sixth, the mortality rate of patients in this study was relatively low (<7%). This may be related to the specificity of the population. In this study, the vast majority of patients came from TCM hospitals. In the perception of Chinese patients, TCM is more appropriate for cardiac rehabilitation. As a result, the majority of patients who sought treatment in TCM hospitals had milder illnesses, and Table 1 shows that patients with severe HF accounted for only around 15% of total hospital admissions. The specificity of the population therefore limits the generalizability of our findings.

## Conclusion

In short, an AoD greater than 37 mm is indeed a dangerous threshold for Chinese HF patients because higher AoD is associated with a higher risk of 12-month mortality above this threshold.

## Data availability statement

The raw data supporting the conclusions of this article will be made available by the authors, without undue reservation.

## Author contributions

G-ZC and TZ: conception or design of the work, data analysis and interpretation, critical revision of the article, and final approval of the version to be published. JC and Y-LH: data collection and drafting the article. HH: data collection and revision of the article. All authors contributed to the article and approved the submitted version.

## Footnotes

1
http://www.R-project.org

2
http://www.empowerstats.com

## Supplementary material

Supplementary Figure 1 The non-linear relationship between AoD and 12-month mortality of HF **(A,B)**. **(A,B)** Demonstrate trends in AoD and death across gender, using 12-month cardiac death as the outcome variable. **(C,D)** Show trends in AoD vs. death in people with different heart failure types using 12-month all-cause death as the outcome variable. The different patterns of lines represent different subgroups of the population.
