# Supplementary material for: The non-linear association between ascending aorta diameter and risk of 12-month mortality in Chinese patients with heart failure: A retrospective cohort study
Source: Front Cardiovasc Med. 2022 Aug 30;9:917325. doi: 10.3389/fcvm.2022.917325 (PMC9468420; doi:10.3389/fcvm.2022.917325)
Supplement: Supplementary file 1 [file Data_Sheet_1.docx]

Supplemental table 1: Baseline characteristics of patients between missing-AoD and non-missing AoD

|  | non-missing AoD | Missing-AoD | P-value |
| --- | --- | --- | --- |
| N | 521 | 54 |  |
| Age, mean±sd，year | 71.97 ± 12.61 | 67.47 ± 18.39 | 0.268 |
| Ln hs-Tnl , mean±sd, per 1 SD change | 0.00 ± 1.00 | -0.06 ± 0.98 | 0.728 |
| Ln Nt-Pro-BNP, mean±sd, per 1 SD change | -0.02 ± 0.99 | 0.09 ± 1.03 | 0.518 |
| BMI, mean±sd, kg/m^2^ | 23.43 ± 3.52 | 23.24 ± 2.28 | 0.697 |
| Sex, No(%) |  |  | 0.459 |
| female | 271 (52.12%) | 31 (57.41%) |  |
| male | 249 (47.88%) | 23 (42.59%) |  |
| NYHA classification on admission, No(%) |  |  | 0.573 |
| Ⅱ+Ⅲ | 447 (86.13%) | 48 (88.89%) |  |
| Ⅳ | 72 (13.87%) | 6 (11.11%) |  |
| Comorbidity-Hypertension, No(%) |  |  | 0.775 |
| No | 139 (27.20%) | 12 (29.27%) |  |
| Yes | 372 (72.80%) | 29 (70.73%) |  |
| Comorbidity-Diabetes, No(%) |  |  | 0.678 |
| No | 384 (75.15%) | 32 (78.05%) |  |
| Yes | 127 (24.85%) | 9 (21.95%) |  |
| Comorbidity-ischemic etiology, No(%) |  |  | 0.038 |
| No | 186 (35.70%) | 27 (50.00%) |  |
| Yes | 335 (64.30%) | 27 (50.00%) |  |
| Comorbidity-COPD, No(%) |  |  | 0.163 |
| No | 449 (87.87%) | 39 (95.12%) |  |
| Yes | 62 (12.13%) | 2 (4.88%) |  |
| Heart Valve Diseases, No(%) |  |  | 0.421 |
| No | 457 (89.43%) | 35 (85.37%) |  |
| Yes | 54 (10.57%) | 6 (14.63%) |  |
| Diuretics use, No(%) |  |  | 0.344 |
| No | 134 (26.22%) | 8 (19.51%) |  |
| Yes | 377 (73.78%) | 33 (80.49%) |  |
| Digoxin use, No(%) |  |  | 0.783 |
| No | 454 (88.85%) | 37 (90.24%) |  |
| Yes | 57 (11.15%) | 4 (9.76%) |  |
| Statins use, No(%) |  |  | 0.610 |
| No | 143 (27.98%) | 13 (31.71%) |  |
| Yes | 368 (72.02%) | 28 (68.29%) |  |
| Sacubitril Trigesartan use, No(%) |  |  | 0.459 |
| No | 433 (83.11%) | 47 (87.04%) |  |
| Yes | 88 (16.89%) | 7 (12.96%) |  |
| Ras Blocker use, No(%) |  |  | 0.018 |
| No | 110 (21.11%) | 19 (35.19%) |  |
| Yes | 411 (78.89%) | 35 (64.81%) |  |
| Heart failure duration, No(%) |  |  | 0.138 |
| 0-1 years | 408 (79.84%) | 31 (75.61%) |  |
| 1-3 years | 47 (9.20%) | 8 (19.51%) |  |
| 3-5years | 34 (6.65%) | 1 (2.44%) |  |
| >5 years | 22 (4.31%) | 1 (2.44%) |  |
| Heart failure classification (by LVEF), No(%) |  |  | 0.683 |
| HFrEF (LVEF < 40%) | 97 (18.62%) | 3 (12.00%) |  |
| HFmrEF (LVEF 40–49%) | 66 (12.67%) | 3 (12.00%) |  |
| HFpEF (LVEF ≥50%) | 358 (68.71%) | 19 (76.00%) |  |

Note: The sum does not match the total sample size due to missing data

Supplemental table 2: Baseline characteristics of patients between Left-censored patient and non-Left-censored patient

|  | non-Left-censored | Left-censored | P-value |
| --- | --- | --- | --- |
| N | 478 | 43 |  |
| Age, mean±sd，year | 72.00 ± 12.68 | 71.60 ± 11.87 | 0.843 |
| Ln hs-Tnl , mean±sd, per 1 SD change | -0.00 ± 1.00 | 0.09 ± 0.99 | 0.556 |
| Ln Nt-Pro-BNP, mean±sd, per 1 SD change | -0.00 ± 0.99 | -0.18 ± 0.97 | 0.273 |
| BMI, mean±sd, kg/m^2^ | 23.45 ± 3.59 | 23.16 ± 2.55 | 0.610 |
| Sex |  |  | 0.595 |
| female | 247 (51.77%) | 23 (56.10%) |  |
| male | 231 (48.23%) | 18 (43.90%) |  |
| NYHA classification on admission |  |  | 0.461 |
| Ⅱ+Ⅲ | 410 (85.80%) | 36 (90.00%) |  |
| Ⅳ | 68 (14.20%) | 4 (10.00%) |  |
| Comorbidity-Hypertension |  |  | 0.215 |
| No | 131 (27.93%) | 8 (19.05%) |  |
| Yes | 337 (72.07%) | 34 (80.95%) |  |
| Comorbidity-Diabetes |  |  | 0.363 |
| No | 350 (74.63%) | 34 (80.95%) |  |
| Yes | 119 (25.37%) | 8 (19.05%) |  |
| Comorbidity-ischemic etiology |  |  | 0.738 |
| No | 172 (35.91%) | 14 (33.33%) |  |
| Yes | 306 (64.09%) | 28 (66.67%) |  |
| Comorbidity-COPD |  |  | 0.301 |
| No | 410 (87.42%) | 39 (92.86%) |  |
| Yes | 59 (12.58%) | 3 (7.14%) |  |
| Heart Valve Diseases |  |  | 0.769 |
| No | 420 (89.55%) | 37 (88.10%) |  |
| Yes | 49 (10.45%) | 5 (11.90%) |  |
| Diuretics use |  |  | 0.467 |
| No | 121 (25.80%) | 13 (30.95%) |  |
| Yes | 348 (74.20%) | 29 (69.05%) |  |
| Digoxin use |  |  | 0.726 |
| No | 416 (88.70%) | 38 (90.48%) |  |
| Yes | 53 (11.30%) | 4 (9.52%) |  |
| Statins use |  |  | 0.787 |
| No | 132 (28.14%) | 11 (26.19%) |  |
| Yes | 337 (71.86%) | 31 (73.81%) |  |
| Sacubitril Trigesartan use |  |  | 0.093 |
| No | 401 (83.92%) | 31 (73.81%) |  |
| Yes | 77 (16.08%) | 11 (26.19%) |  |
| Ras Blocker use |  |  | 0.655 |
| No | 100 (20.88%) | 10 (23.81%) |  |
| Yes | 378 (79.12%) | 32 (76.19%) |  |
| Heart failure duration |  |  | 0.028 |
| 0-1 years | 366 (78.25%) | 41 (97.62%) |  |
| 1-3 years | 46 (9.81%) | 1 (2.38%) |  |
| 3-5years | 34 (7.25%) | 0 (0.00%) |  |
| >5 years | 22 (4.69%) | 0 (0.00%) |  |
| Heart failure classification (by LVEF) |  |  | 0.595 |
| HFrEF (LVEF < 40%) | 91 (19.00%) | 6 (14.29%) |  |
| HFmrEF (LVEF 40–49%) | 59 (12.32%) | 7 (16.67%) |  |
| HFpEF (LVEF ≥50%) | 328 (68.68%) | 29 (69.05% |  |

Note: The sum does not match the total sample size due to missing data

Supplemental table 3: Covariates screening by >10% effect on the regression coefficient of AoD

| Covariates | basic model | full mode | Selection result |
| --- | --- | --- | --- |
|  | -0.0144# | -0.0196# |  |
| Age | -0.0142 | -0.0198 |  |
| Sex | -0.0126 * | -0.0147 * | Yes |
| NYHA classification | -0.0201 * | -0.0053 * | Yes |
| Hypertension | -0.0100 * | -0.0200 | Yes |
| Diabetes | -0.0152 | -0.0188 |  |
| Ischemic etiology | -0.0134 | -0.0197 |  |
| COPD | -0.0129 * | -0.0190 | Yes |
| Heart Valve Diseases | -0.0148 | -0.0170 * | Yes |
| Diuretics use | -0.0163 * | -0.0195 | Yes |
| Digoxin use | -0.0175 * | -0.0122 * | Yes |
| Statins use | -0.0157 | -0.0072 * | Yes |
| Sacubitril Trigesartan use | -0.0109 * | -0.0188 | Yes |
| Heart failure duration | -0.0055 * | -0.0299 * | Yes |
| Ln Nt-Pro-BNP z score | -0.0057 * | -0.0216 * | Yes |
| Ln hs-Tnl Z score | -0.0144 | -0.0193 |  |
| Ras-blocker use | -0.0114 * | -0.0221 * | Yes |
| BMI | -0.0204 * | -0.0116 * | Yes |
| Heart failure classification (by LVEF) | 0.0044 * | -0.0280 * | Yes |

#: starting regression coefficients;

* Indicates a change of more than 10% from the starting regression coefficient

Supplemental Table 4: Subgroup analyses stratified by sex

| Stratified variable | Female  OR, 95%CI | Male  OR, 95%CI |
| --- | --- | --- |
| Fitting using standard binary logistic regression model | 0.82 (0.64, 1.04) | 1.04 (0.86, 1.24) |
| Fitting using two piecewise linear model |  |  |
| Ascending aorta diameter (mm) Inflection poit | 37 | 37 |
| <= Inflection poit | 0.82 (0.64, 1.06) | 0.81 (0.59, 1.11) |
| > Inflection poit | 0.01 (0.00, Inf) | 1.67 (0.99, 3.10) |
| P for log-likely hood ratio test | 0.780 | 0.011 |

Inf means model failed due to small sample size

Covariates which were adjusted for: Sex, NYHA classification on admission, hypertension, COPD, Heart Valve Diseases, diuretics use, digoxin use, statins use, sacubitril trigesartan use, heart failure duration, Ln Nt-Pro-BNP (z-score), Ras blocker use, BMI, Heart failure classification

Supplemental Table 5: Subgroup analyses stratified by LVEF

| LVEF(%) | HFrEF and HFmrEF  OR, 95%CI | HFpEF  OR, 95%CI |  |
| --- | --- | --- | --- |
| Fitting using standard binary logistic regression model | 1.12 (0.97, 1.29) | 0.90 (0.77, 1.04) |  |
| Fitting using two piecewise linear model |  |  |  |
| Ascending aorta diameter (mm) Inflection poit | 37 | 37 |  |
| <= Inflection poit | 0.99 (0.79, 1.24) | 0.91 (0.78, 1.07) |  |
| > Inflection poit | 1.35 (0.99, 1.84) | 0.00 (0.00, Inf) |  |
| P for log-likely hood ratio test | 0.048 | 0.479 |  |

Inf: model is failed due to small sample size

Covariates which were adjusted for: Sex, NYHA classification on admission, hypertension, COPD, Heart Valve Diseases, diuretics use, digoxin use, statins use, sacubitril trigesartan use, heart failure duration, Ln Nt-Pro-BNP (z-score), Ras blocker use, BMI, Heart failure classification
