# Supplementary figures and images for: The non-linear association between ascending aorta diameter and risk of 12-month mortality in Chinese patients with heart failure: A retrospective cohort study
Source: Front Cardiovasc Med. 2022 Aug 30;9:917325. doi: 10.3389/fcvm.2022.917325 (PMC9468420; doi:10.3389/fcvm.2022.917325)

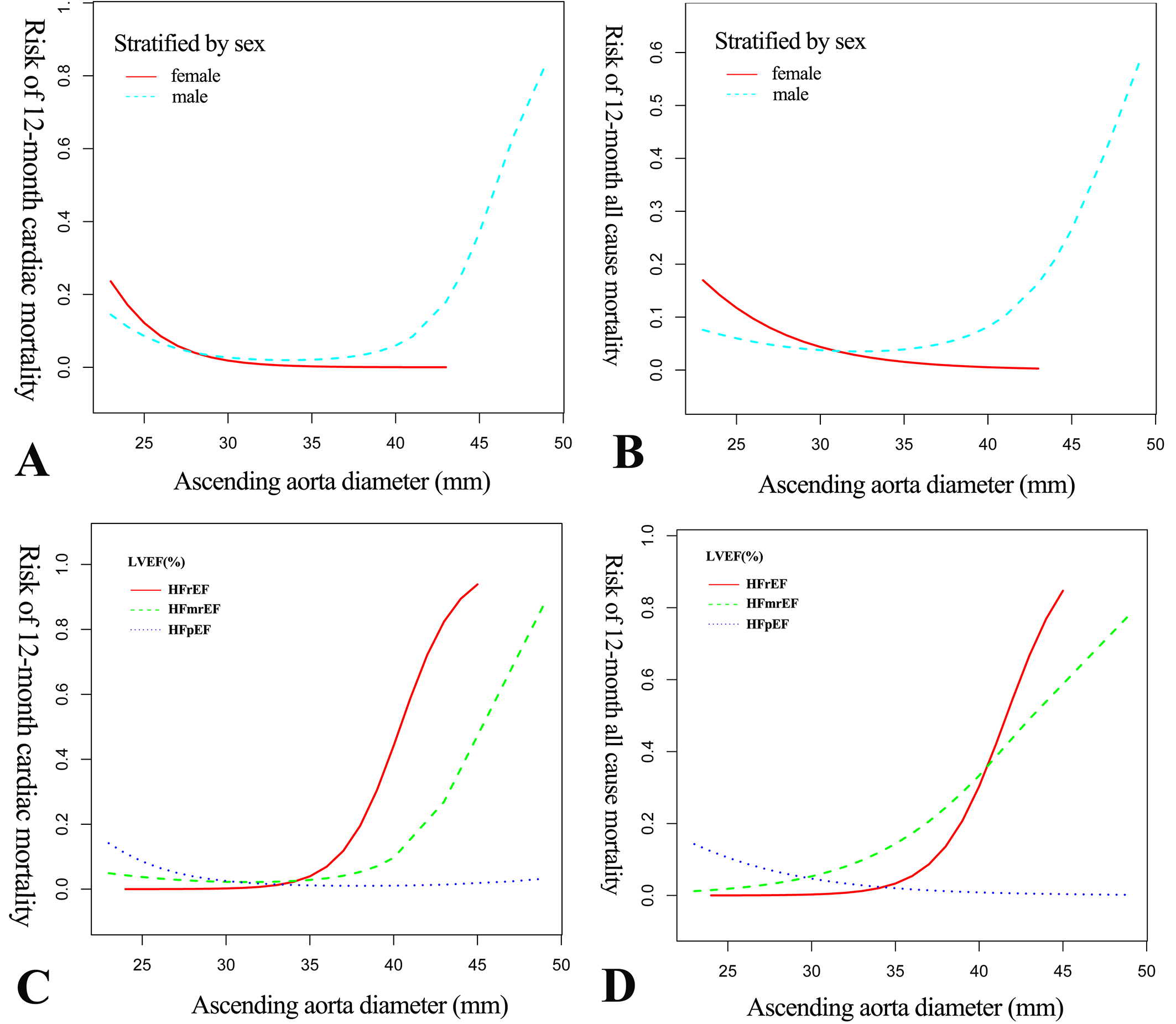

Supplement: Supplementary Figure 1 — The non-linear relationship between AoD and 12-month mortality of HF (A,B). (A,B) Demonstrate trends in AoD and death across gender, using 12-month cardiac death as the outcome variable. (C,D) Show trends in AoD vs. death in people with different heart failure types using 12-month all-cause death as the outcome variable. The different patterns of lines represent different subgroups of the population. [file Image_1.TIF]
